# Supplementary material for: A recurrent cancer-associated substitution in DNA polymerase ε produces a hyperactive enzyme
Source: Nat Commun. 2019 Jan 22;10:374. doi: 10.1038/s41467-018-08145-2 (PMC6343027; doi:10.1038/s41467-018-08145-2)
Supplement: Supplementary file 1 — Supplementary Information [file 41467_2018_8145_MOESM1_ESM.pdf]

SUPPLEMENTARY MATERIAL

A recurrent cancer-associated substitution in DNA polymerase ε produces a hyperactive enzyme

Xing et al.

SUPPLEMENTARY METHODS

Active site titration

We used a modification of the previously described procedure[1] to compare the fraction of active polymerase in the preparations of wild-type Polε, exo$^-$ Polε and Polε-P301R. For each reaction, a 7-μl mixture of Cy5-labelled P50/T80 substrate, dNTPs, and Polε in the reaction buffer was first incubated at 30°C for 5 min to form DNA-Polε complex. A 5-μl mixture of single-stranded M13mp2 DNA and $MgAc_2$ was then added, and DNA synthesis was allowed to proceed for 10 min at 30°C. The single-stranded M13mp2 DNA acts as a trap to capture any dissociating Polε molecules and ensure single-hit conditions of the polymerase reaction[2]. The final 12-μl reactions contained 33 mM Tris-HCl pH 7.8, 0.8 mM dithiothreitol, 0.17 mg/ml bovine serum albumin, 8 mM $MgAc_2$, 104 mM NaAc, 83 nM Cy5-labelled P50/T80, 830 nM single-stranded M13mp2, dNTPs at the intracellular S-phase concentrations (30 μM dCTP, 80 μM dTTP, 38 μM dATP, and 26 μM dGTP) and Polε at increasing concentrations as indicated. To confirm that polymerase could not re-associate with the P50/T80 substrate under these conditions, a control reaction was performed as follows. The single-stranded M13mp2 was preincubated with Polε and dNTPs in the reaction buffer at 30°C for 5 min in a 9-ul reaction, and a 3-μl mixture of Cy5-labelled P50/T80 substrate and $MgAc_2$ was then added, followed by incubation at 30°C for 10 min. The absence of detectable DNA synthesis in the control reaction confirmed efficient trapping of free Polε molecules. The reactions were quenched by the addition of an equal volume of 2x loading buffer containing 95% deionized formamide, 100 mM EDTA, and 0.025% Orange G. After

boiling for 3 min and cooling on ice, 6-μl samples were subjected to electrophoresis in 10% denaturing polyacrylamide gel containing 8 M urea in 1x TBE. Quantification was done by fluorescence imaging on a Typhoon system (GE Healthcare).

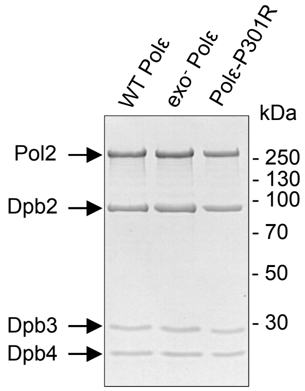

Supplementary Figure 1. SDS PAGE analysis of purified wild-type Polε, proofreading-deficient Polε and Polε-P301R. The proteins (5 µg in each lane) were separated in a 4-12% Bis-Tris gel and visualized by Coomassie staining. WT Polε, wild-type Polε. exo$^-$ Polε, proofreading-deficient Polε. Uncropped gel image is provided in a Source Data file.

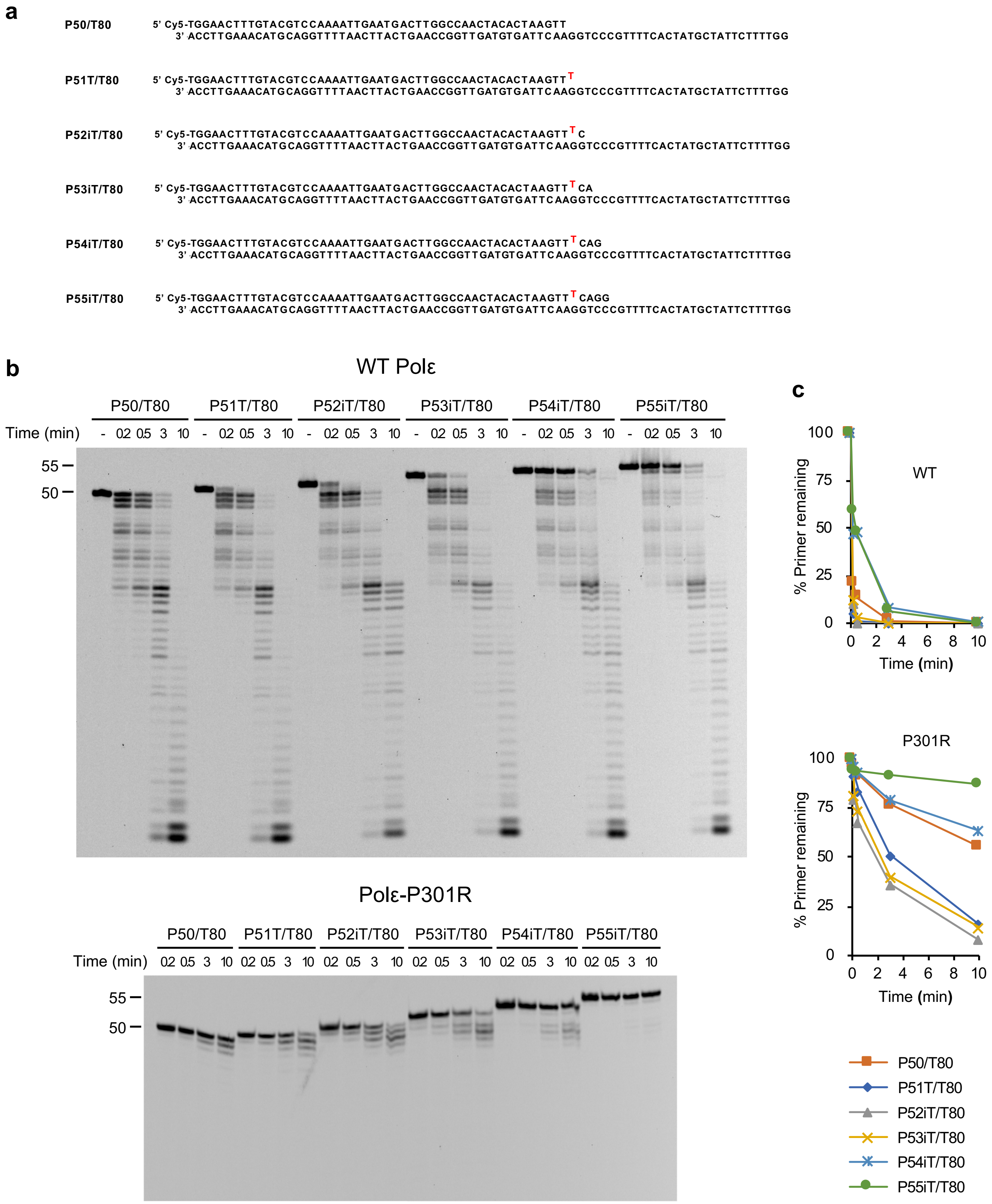

Supplementary Figure 2. Exonuclease activity of wild-type Polε (WT) and Polε-P301R on matched and mismatched DNA. (**a**) DNA substrates. The mismatched base is in red. (**b**) Exonuclease activity was assayed as in Fig. 1a. (**c**) Quantification of (b). The activity of both enzymes is maximal when a mismatch is present within 3 bp from the 3' end. Reduced activity with P54iT/T80 and P55iT/T80 in comparison with P50/T80 likely reflects a stabilizing effect of terminal G-C pairs that are not present in P50/T80. Representative of three independent experiments. Source data for (b) and (c) are provided in a Source Data file.

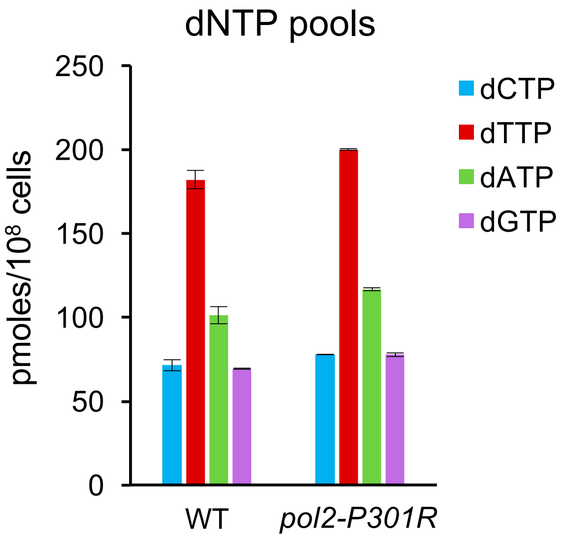

Supplementary Figure 3. Intracellular dNTP levels in wild-type and *pol2-P301R* strains. The dNTP concentrations were measured in asynchronous logarithmic phase cultures as described previously[17]. Data are presented as the mean for two independent measurements performed with the wild-type strain and two independent *pol2-P301R* isolates. Error bars show the range of values. Source data are provided in a Source Data file.

a exo⁻ Polε

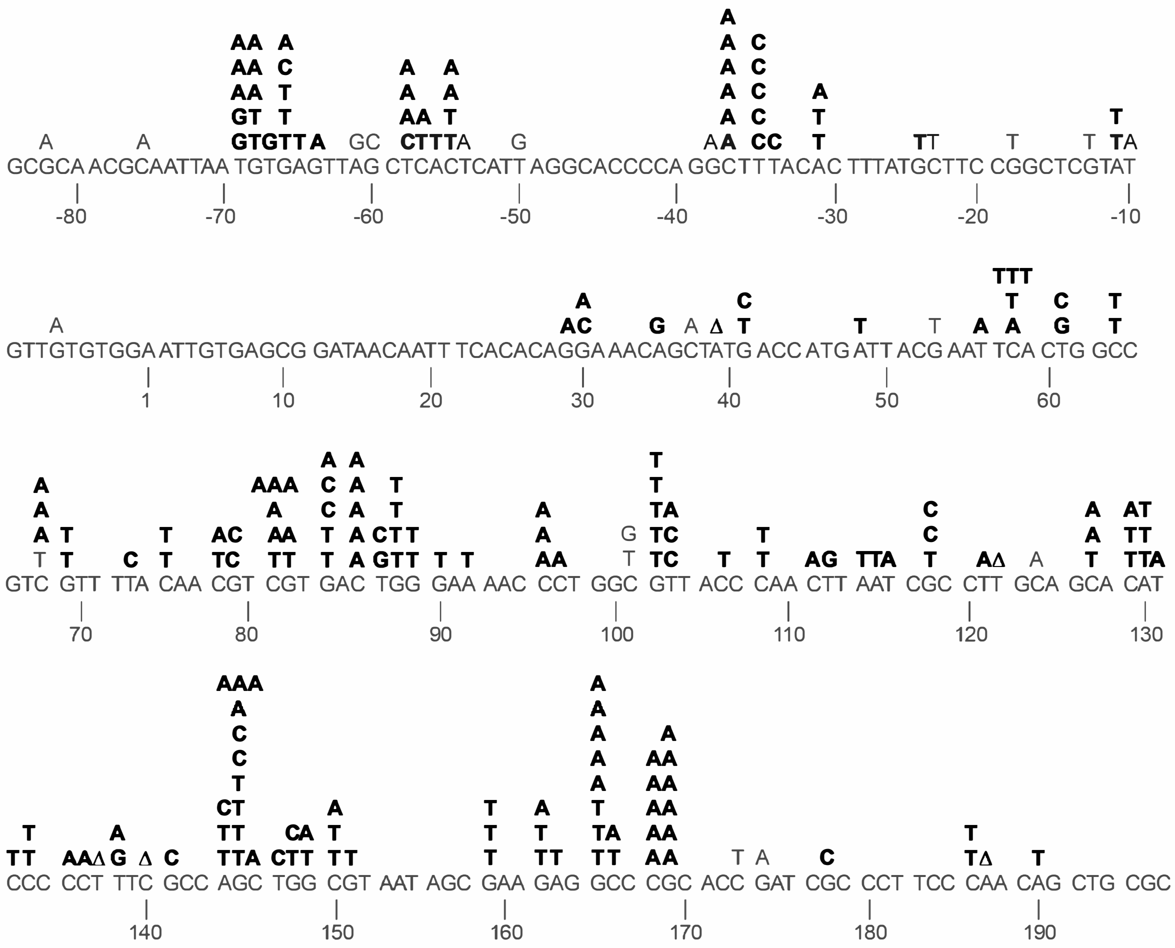

Supplementary Figure 4. (continued)

b Polε-P301R

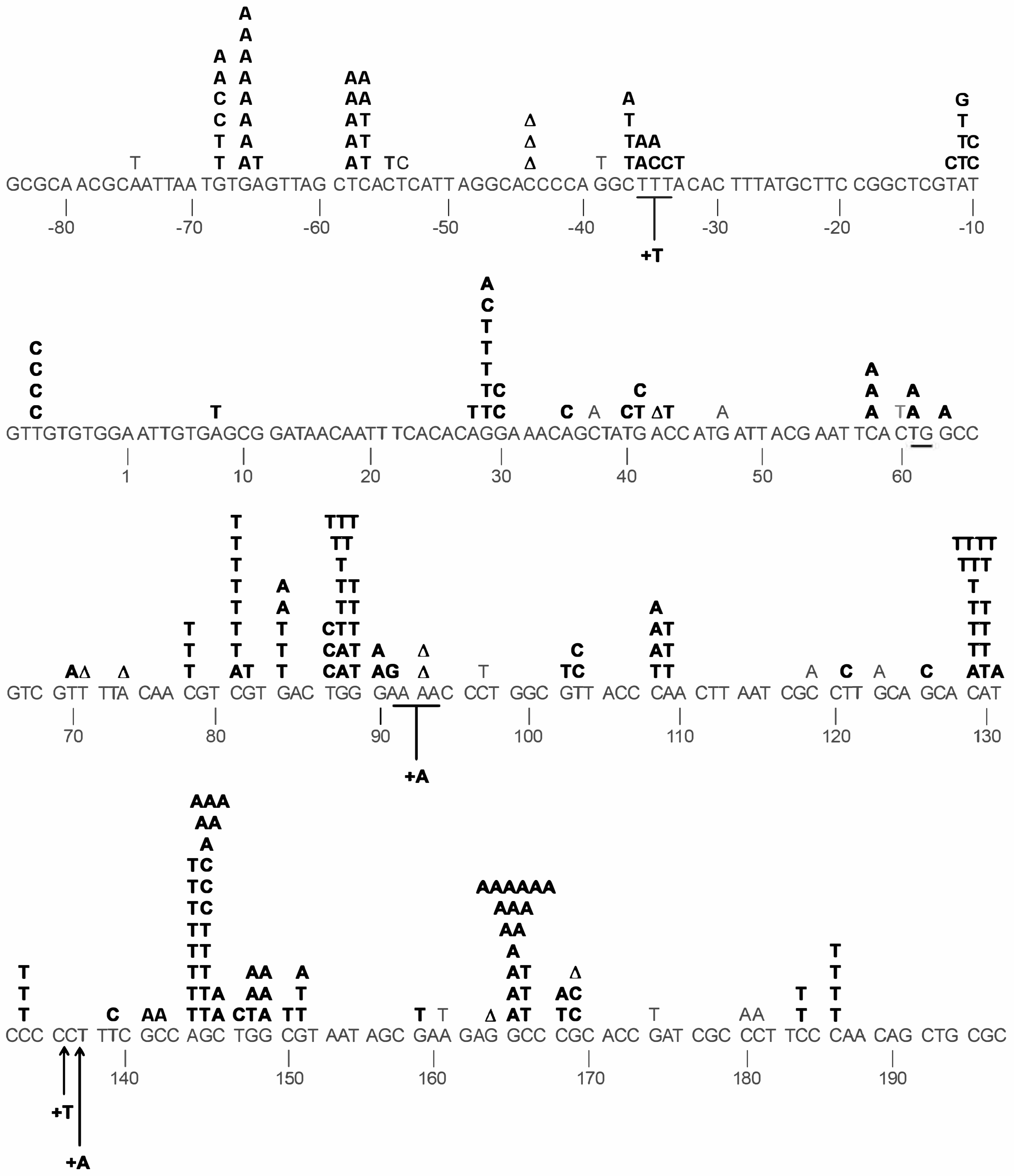

Supplementary Figure 4. Spectra of single-base substitutions and insertion/deletion mutations generated by exo[-] Polε (a) and Polε-P301R (b) in the *lacZ* gene. Base substitutions and single-base deletions (open triangles) are shown above the *lacZ* sequence. Insertions are under the *lacZ* sequence. Deletion of two base pairs is shown as a line under the *lacZ* sequence. Detectable mutations are in black, bold text. Silent mutations are in plain, gray text.

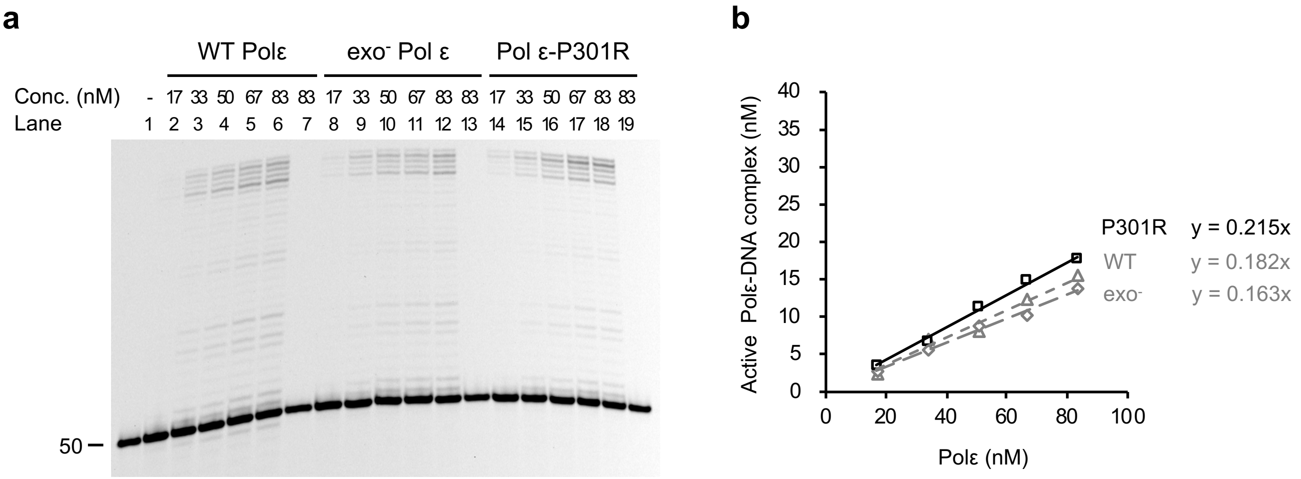

Supplementary Figure 5. Active site titration of wild-type (WT) Polε, exo$^-$ Polε and Polε-P301R. (**a**) *Lanes 2-6, 8-12 and 14-18*: Polε variants (0.2 – 1 pmol) were incubated with 1 pmol of Cy5-labelled P50/T80 and dNTP at 30°C for 5 min to form DNA-Polε-dNTP ternary complex, and $MgAc_2$ was then added along with 10 pmol of single-stranded M13mp2 trap to allow primer extension under single-hit conditions. Negative controls in *lanes 7, 13 and 19* confirmed the effectiveness of M13mp2 trap: 1 pmol of Polε was incubated with 10 pmol M13mp2 prior to the addition of $MgAc_2$ and Cy5-labelled P50/T80, and no extension of the Cy5-labeled primer was observed. *Lane 1*: P50/T80 DNA substrate alone. See Supplementary Methods for details. (**b**) Quantification of active fraction of Polε variants. The concentration of active Polε-DNA complexes plotted on the Y axis was calculated by multiplying the fraction of extended primer by the total P50/T80 concentration in the reaction. The concentration of Polε present in the reaction is plotted along the X axis. The slope of the line shows the active fraction of the protein preparations. Note that this represents a minimum estimate of the active enzyme fraction, since some molecules could dissociate before engaging in DNA synthesis. While the active fractions are generally similar between wild-type Polε, exo$^-$ Polε and Polε-P301R preparations, a slightly higher estimate for Polε-P301R could conceivably reflect its reduced propensity to dissociate predicted by our model (Fig. 6). Representative of three independent experiments. Source data are provided in a Source Data file.

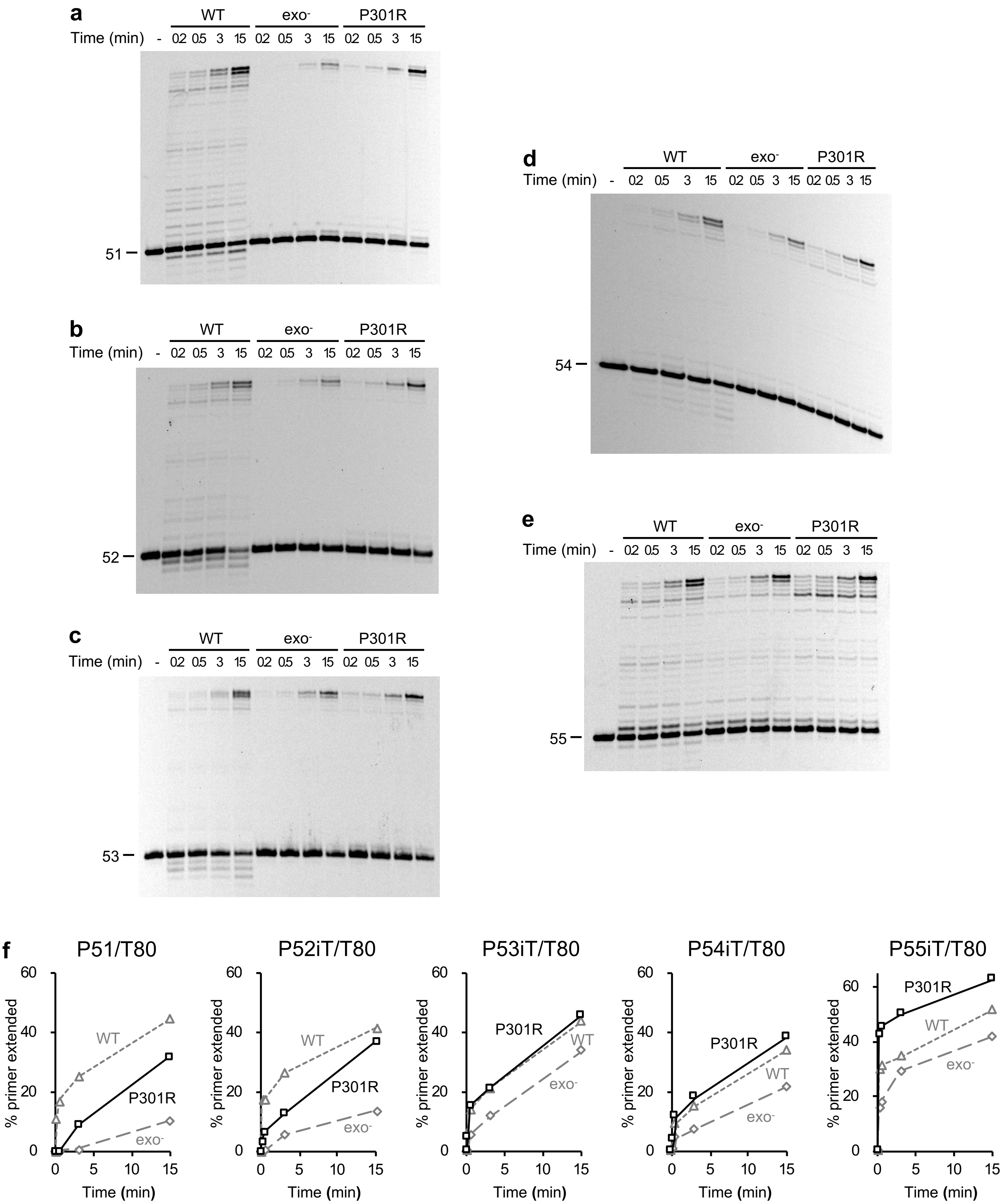

Supplementary Figure 6. Extension of primers with terminal and internal mismatches by wild-type Polε (WT), exo⁻ Polε and Polε-P301R. The mismatched substrates are described in Supplementary Fig. 2a. Polymerase activity was assayed as in Fig. 4a with 0.8 nM Polε and 25 nM P51T/T80 (**a**), P52iT/T80 (**b**), P53iT/T80 (**c**), P54iT/T80 (**d**) and P55iT/T80 (**e**). (**f**) Quantification of (**a-e**). Each panel is representative of two to seven experiments. Source data are provided in a Source Data file.

Supplementary Table 1. Fidelity of *in vitro* DNA synthesis by exo$^-$ Polε and Polε-P301R.

	exo$^-$ Polε		Polε-P301R	
	No.	ER ($x10^{-6}$)[a]	No.	ER ($x10^{-6}$)[a]
Base substitutions	200	155	208	59
Transitions	69	144	110	84
A→G (A-dCTP)	1	8.5	2	6.2
G→A (G-dTTP)	26	236	46	153
C→T (C-dATP)	29	239	44	133
T→C (T-dGTP)	13	101	18	51
Transversions	131	162	98	44
A→C (A-dGTP)	1	11	1	4.0
A→T (A-dATP)	15	110	22	59
C→A (C-dTTP)	42	357	15	47
C→G (C-dCTP)	0	<15	0	<5.7
G→C (G-dGTP)	14	127	12	40
G→T (G-dATP)	40	351	36	116
T→A (T-dTTP)	12	150	12	55
T→G (T-dCTP)	7	74	0	<3.9
Single-base indels	5	3.2	15	3.3
Minus 1	5	6.5	10	4.8
Plus 1	0	<1.3	4	1.9
Other			1	
Total	205		222	
*lac*Z mutant frequency	0.032		0.012	

The data are based on analysis of 205 and 212 *lac*Z mutants created by exo$^-$ Polε and Polε-P301R, respectively. 21 of 205 mutants created by exo$^-$ Polε contained more than one mutation, and 21 of 212 mutants created by Polε-P301R contained more than one mutation. Only phenotypically detectable *lac*Z mutations are listed. The location of individual mutations in the *lac*Z sequence is shown in Supplementary Fig. 4. The background mutant frequency for unfilled M13mp2 gapped substrate was 0.00062.

[a]Error rates (ER) for individual mutation types were calculated as described in Methods. Silent mutations were excluded from the ER calculation.

Supplementary Table 2. Spontaneous *can1* mutations in *pol2-P301R* yeast strain.

Mutation	No. of detectable mutations
Base substitutions	
GC to AT	60
GC to TA	49
GC to CG	1
AT to GC	10
AT to CG	4
TA to TA	21
Single-base indels	
Minus 1	9
Plus 1	45
Total	199

The data are based on DNA sequence analysis of 194 Can^r mutants containing a total of 199 mutations in the *CAN1* gene.

SUPPLEMENTARY REFERENCES

1. Cai, H., Yu, H., McEntee, K., Kunkel, T.A. & Goodman, M.F. Purification and properties of wild-type and exonuclease-deficient DNA polymerase II from *Escherichia coli*. *J Biol Chem* **270**, 15327-35 (1995).
2. Maki, S., Hashimoto, K., Ohara, T. & Sugino, A. DNA polymerase II (ε) of *Saccharomyces cerevisiae* dissociates from the DNA template by sensing single-stranded DNA. *J Biol Chem* **273**, 21332-41 (1998).
3. Mertz, T.M., Sharma, S., Chabes, A. & Shcherbakova, P.V. Colon cancer-associated mutator DNA polymerase δ variant causes expansion of dNTP pools increasing its own infidelity. *Proc Natl Acad Sci USA* **112**, E2467-76 (2015).
