# Supplementary material for: A recurrent cancer-associated substitution in DNA polymerase ε produces a hyperactive enzyme
Source: Nat Commun. 2019 Jan 22;10:374. doi: 10.1038/s41467-018-08145-2 (PMC6343027; doi:10.1038/s41467-018-08145-2)
Supplement: Supplementary file 4 — Reporting summary [file 41467_2018_8145_MOESM4_ESM.pdf]

nature research

Corresponding author(s): Polina V. Shcherbakova

Reporting Summary

Nature Research wishes to improve the reproducibility of the work that we publish. This form provides structure for consistency and transparency in reporting. For further information on Nature Research policies, see Authors & Referees and the Editorial Policy Checklist.

Statistical parameters

When statistical analyses are reported, confirm that the following items are present in the relevant location (e.g. figure legend, table legend, main text, or Methods section).

n/a	Confirmed	
☐	☒	The exact sample size (*n*) for each experimental group/condition, given as a discrete number and unit of measurement
☐	☒	An indication of whether measurements were taken from distinct samples or whether the same sample was measured repeatedly
☐	☒	The statistical test(s) used AND whether they are one- or two-sided<br>*Only common tests should be described solely by name; describe more complex techniques in the Methods section.*
☒	☐	A description of all covariates tested
☒	☐	A description of any assumptions or corrections, such as tests of normality and adjustment for multiple comparisons
☐	☒	A full description of the statistics including central tendency (e.g. means) or other basic estimates (e.g. regression coefficient) AND variation (e.g. standard deviation) or associated estimates of uncertainty (e.g. confidence intervals)
☐	☒	For null hypothesis testing, the test statistic (e.g. *F*, *t*, *r*) with confidence intervals, effect sizes, degrees of freedom and *P* value noted<br>*Give P values as exact values whenever suitable.*
☒	☐	For Bayesian analysis, information on the choice of priors and Markov chain Monte Carlo settings
☒	☐	For hierarchical and complex designs, identification of the appropriate level for tests and full reporting of outcomes
☒	☐	Estimates of effect sizes (e.g. Cohen's *d*, Pearson's *r*), indicating how they were calculated
☐	☒	Clearly defined error bars<br>*State explicitly what error bars represent (e.g. SD, SE, CI)*

Our web collection on statistics for biologists may be useful.

Software and code

Policy information about availability of computer code

Data collection	No software was used.
Data analysis	No software was used.

For manuscripts utilizing custom algorithms or software that are central to the research but not yet described in published literature, software must be made available to editors/reviewers upon request. We strongly encourage code deposition in a community repository (e.g. GitHub). See the Nature Research guidelines for submitting code & software for further information.

Data

Policy information about availability of data

All manuscripts must include a data availability statement. This statement should provide the following information, where applicable:

- Accession codes, unique identifiers, or web links for publicly available datasets
- A list of figures that have associated raw data
- A description of any restrictions on data availability

All data used to reach the conclusions are presented fully within the article and the supplementary material.

Field-specific reporting

Please select the best fit for your research. If you are not sure, read the appropriate sections before making your selection.

☒ Life sciences ☐ Behavioural & social sciences ☐ Ecological, evolutionary & environmental sciences

For a reference copy of the document with all sections, see nature.com/authors/policies/ReportingSummary-flat.pdf

Life sciences study design

All studies must disclose on these points even when the disclosure is negative.

Sample size	For mutation rate analysis, the use of nine independent cultures is necessary and usually sufficient to demonstrate 2-fold and higher differences in the mutation rate at 95% confidence level. No sample size calculations were performed for the mutation spectra analysis, because sample size required for comparison of the proportion of individual mutations depends on the mutation spectrum itself, which is not known prior to the experiment. in our prior experience, a collection of 200-250 mutations allows us to detect differences in the proportions of major mutational classes at >95% confidence level.
Data exclusions	No data were excluded from the analysis.
Replication	All DNA polymerase and exonuclease assays were performed at least three times. The mutation rates were measured using at least two independently constructed strains of each genotype. All reported results were reproducible.
Randomization	Not relevant to the present work. The results were compared for the wild-type, D290A/E292A and P301R derivatives of DNA polymerase epsilon, the parameters fully controlled in our experiments.
Blinding	The investigators were not blinded during data collection and analysis. Each part of the work was performed by a single investigator, as described in the author contribution section, and knowing which polymerase variant is being used in the reaction was essential for the proper execution of the experiments and their interpretation. All data collected are objective measurements not subject to investigator bias.

Reporting for specific materials, systems and methods

Materials & experimental systems

n/a	Involved in the study
☐	☒ Unique biological materials
☒	☐ Antibodies
☒	☐ Eukaryotic cell lines
☒	☐ Palaeontology
☒	☐ Animals and other organisms
☒	☐ Human research participants

Methods

n/a	Involved in the study
☒	☐ ChIP-seq
☒	☐ Flow cytometry
☒	☐ MRI-based neuroimaging

Unique biological materials

Policy information about availability of materials

Obtaining unique materials	All yeast strains and plasmids used or created in this work are freely available from the authors upon request.
